# Challenges and opportunities in microRNA-based cancer therapeutics

**DOI:** 10.1016/j.xcrm.2025.102057

**Published:** 2025-04-15

**Authors:** Jingfang Ju

**Affiliations:** 1 Department of Pathology, Stony Brook Cancer Center, Renaissance School of Medicine, Stony Brook, NY 11794-8691, USA

## Abstract

The 2024 Nobel Prize honored groundbreaking microRNA (miRNA) discoveries that unveiled the critical functions of miRNAs in fundamental biology and human health. Despite promising therapeutic potential, there are no Food and Drug Administration (FDA)-approved miRNA-based cancer therapies. This commentary discusses the progress and challenges of miRNA-based cancer therapeutics and their potential impact on future clinical oncology.

## Main text

The human genome is rather complex, with less than 2% of protein-coding genes making up the genome. Over 98% of the human genome is non-coding RNAs. MicroRNAs (miRNAs) are a class of non-coding RNAs with the crucial function of regulating gene expression. miRNAs, the 2024 Nobel Prize award-winning discovery, were first discovered in *Caenorhabditis elegans* in 1993.^1^^,^^2^ A decade later, it was discovered that miRNAs impact cancer development, as deletion of miR-15 and miR-16 is directly linked to chronic lymphocytic leukemia.^3^ Since then, the field of miRNA cancer research has grown exponentially over the last two decades to provide a better understanding of the mechanisms of miRNA in cancer biology. These efforts establish the foundation for miRNA-based cancer therapeutics.

### Understanding miRNA biogenesis and function in cancer

miRNAs are small (20–22 nucleotides) double-stranded, non-coding RNA molecules that are processed from larger pre-miRNAs by the RNase III enzyme Dicer (DICER1).^4^ One strand of the miRNA duplex is loaded into an RNA-induced silencing complex (RISC) while the other strand is degraded by cellular nucleases.^5^ The miRNA-RISC complex binds to specific messenger RNA (mRNA) targets, leading to translational repression, deadenylation, or cleavage of these mRNAs. Most mammalian miRNAs are thought to repress target gene expression at the translational level^1^^,^^6^^,^^7^ via imperfect base-pairing to the 3′-untranslated regions (3′-UTRs) of their target mRNAs. This translational regulation provides the cell with a precise, immediate, and energy-efficient way of controlling the expression of a given protein. Additionally, translational control of gene expression is readily reversible, providing the cell with great flexibility in responding to various cytotoxic and genotoxic stresses. Individual miRNAs can regulate the expression of multiple target genes via imperfect base-pairing. Unsurprisingly, miRNAs have been shown to have important roles in cancer, regulating critical cancer-related cellular pathways (e.g., Myc, AKT, Wnt, Hippo, and mTOR) and exhibiting dual functionality as tumor suppressors or oncogenes depending on the target repertoire. Tumor-suppressive miRNAs particularly demonstrate therapeutic promise through their capacity for simultaneously targeting multiple cancer-driving pathways, overcoming the limitations of single-target therapies. Cancer treatment regimens have advanced with the discovery of novel detection methods and therapeutics. As a result of these advances, cancer mortality rates have continued to improve. Unfortunately, despite these developments, mortality rates in those diagnosed with metastatic cancers have remained generally poor due to disease relapse and refractoriness after various treatment regimens. The continuous poor survival rates can be seen in patients with lung cancer, breast cancer, colorectal cancer (CRC), pancreatic cancer, gastric cancer, and liver cancer. One of the major treatment obstacles that has contributed to mortality has been resistance to therapy.

Currently, there are a variety of therapies available for patients with metastatic cancer, including chemotherapy, radiotherapy, targeted therapy, and immunotherapy. Depending on the patient’s condition, these therapies can also be combined to improve efficacy and patient survival. However, despite novel discoveries in these therapies, cancer recurrence and resistance have still been observed. As tumor cells are highly heterogeneous with many genetic and epigenetic alterations, experimental evidence suggests that cancer stem cells (CSCs) are largely responsible for cancer recurrence and resistance to therapy. CSCs are cancer cells with a “stem-like” nature of continuous self-renewal. It is important to note that although they are called CSCs, this term only refers to how this population exhibits characteristics similar to stem cells. CSCs were first discovered in leukemia, and they were discovered to have acquired tumor-initiating capabilities, as seen by their ability to initiate tumor formation in mouse xenografts. After the initial discovery of CSCs in leukemia, CSC populations were also discovered in solid tumor cancer types such as lung cancer, breast cancer, CRC, pancreatic cancer, gastric cancer, and liver cancer. The stem-like nature of CSCs has been found to contribute to tumor growth, recurrence, and resistance to therapeutics. Furthermore, the plasticity of the CSC state mediated by miRNAs has been observed due to the surrounding CSC niche, thus adding an additional obstacle to therapeutic strategies to eliminate resistant CSCs. As a result, a growing amount of research specifically identifies and targets CSCs, including the roles of miRNAs in maintaining tumor stemness and resistance to therapy. In addition to CSCs, certain miRNAs (e.g., miR-320, miR-155, let-7, miR-143/145) influence aspects of the tumor microenvironment, such as immune cells, inflammation, endothelial cells, and cancer-associated fibroblasts (CAFs). Modulation of miRNAs that impact tumor microenvironment will be another important aspect of tumor resistance.

Although cancer is a genetic disease, epigenetic alterations (e.g., methylation, histone modification, and non-coding RNAs) play key roles in tumorigenesis and resistance to various therapeutic modalities. Alterations regulated by miRNAs enhance the ability of tumor cells to adapt to various growth conditions and their unique tumor microenvironment. Cancer cells are also highly adaptable in the face of therapeutic insults. Epigenetic changes allow cancer cells to rapidly adapt to environmental changes, including exposure to chemotherapy and other therapeutic modalities. Cancer cells can adopt various strategies to survive treatment through gene expression alterations. This includes the cancer cell’s ability to resist apoptosis and to activate/suppress autophagy to overcome the challenge of chemotherapy. Novel therapeutic strategies based on miRNA may potentially address the challenges of cancer resistance mechanisms and the rapidly adaptive nature of cancer to traditional single-targeted therapy.

In the past two decades, we have better understood miRNAs’ tumor suppressor and oncogenic roles in cancer by identifying direct targets and impacted pathways.^8^ We have also gained significant insights into various miRNA degradation and modification mechanisms. Resistance to chemotherapy and radiation therapy is one of the major reasons for the failure of clinical cancer management. Many factors contributed to the resistance mechanism, and miRNAs have been shown to play major roles. Post-transcriptional and translational controls mediated by miRNAs are crucial for tumors to develop resistance quickly by modulating protein synthesis in response to genotoxic stresses during anticancer chemotherapy and/or radiation therapy. As a result, modulating miRNA expression may offer new therapeutic strategies to overcome drug resistance in tumors. Chemotherapeutic agents (e.g., 5-fluorouracil [5-FU] and gemcitabine) are ineffective in eliminating the slow proliferating CSCs; thus, novel approaches may help overcome this issue. It is important to discover genes and pathways responsible for the resistance mechanism. Due to the broad influence of miRNAs in gene expression, miRNAs may offer new insights into the resistance mechanism. In some cases, miRNAs may contribute to the development of resistance, while in others, they may help to overcome resistance. Several tumor suppressor miRNA candidates (e.g., miR-15a, miR-34, miR-143, miR-145, miR-200, miR-506, miR-129, and miR-140) have been identified as potential therapeutic strategies in cancer.

### Pioneer efforts of miRNA-based clinical trials in cancer

The advancement in delivery technology and RNA modification strategy has accelerated the miRNA-based cancer medicine to clinical trials. However, early attempts at miRNA-based cancer therapeutics (e.g., MRX34) were terminated due to some severe immune-related adverse effects in phase 1 clinical trials, even though they were based on promising preclinical safety profiles.^9^

To target oncogenic miRNA targets (e.g., miR-10b, miR-155, and miR-221/222) in cancer, Anti-miRs (also known as Antagomirs) have been utilized based on the modification strategies of antisense oligonucleotides (ASOs). The ASO approach has been well developed and established in the past 4 decades using various chemical modifications to enhance stability, deliverability, and improved PK/PD properties. These modifications include phosphorothioate (PS) backbone, 2′-O-methoxyethyl, 2′-O-methyl, 2′-fluoro, 5-methyl pyrimidine, phosphorodiamidate morpholino (PM), peptide nucleic acid, ribose sugar, and 2′-robose substitutions.^10^ Anti-miRs were designed to bind directly to the mature strand of the target oncogenic miRNAs to block the interaction with the target mRNA transcript or bind to the mRNA target sequences that interact with the miRNA. However, a phase 2 clinical trial using anti-miR-155 for treatment of cutaneous T cell lymphoma was terminated without a clear reason.^11^ These early efforts contribute greatly to our understanding of the issues related to miRNA-based cancer therapy.

### Challenges of miRNA-based cancer therapeutics

There are several challenges for miRNA-based cancer therapeutics. Challenges such as stability, toxicity, distribution, specificity to target tumor cells, and immunogenicity are similar to those faced by other nucleic acid-based compounds. With the extensive efforts from academia and industry, several solutions are now available to bring miRNA therapeutics closer to patients. With the issue of stability, extensive efforts have been made to modify miRNAs for enhanced stability by various modifications of nucleotides, such as 2′-O-methyl and 2′-O-methoxyethyl, as well as with 3′-end-cholesterol conjugation and locked nucleic acid.^10^ Such modifications have been demonstrated to enhance anti-miR stability and affinity to mRNA targets. However, care has to be taken to avoid off-target effects due to extensive modifications.

As for the *in vivo* delivery strategies, a number of approaches have been attempted, such as using lipid nanoparticles (LNPs); polymers, such as polyethyleneimine (PEI); polyactic-co-glycolic acid (PLGA); chitosan; and poly-amidoamine (PAMAM), with various surface modifications including polyethylene glycol (PEG) or hyaluronic acid (HA). Lentiviral vectors have been utilized to offer the advantage of stable gene therapy due to their integration into the genome. It has also been observed that lentiviral vectors offer greater transduction efficiency than other viral vectors in lung cancer. However, there remains the concern that viral integration could disrupt the host genome, although some work has indicated that lentiviral integration poses a limited threat to genomic integrity. There is also concern regarding developing an immune response to the lentiviral vector, which could limit the effectiveness of treatment. Other viral vectors for miRNA delivery are adenoviral-based vectors. Adenoviral-based vectors are episomal compared with lentiviral vectors but also have toxicity-related issues associated with immune response to the virus and the concern of overcoming existing immunity. Adeno-associated virus has also been reported to be effective at delivering miRNA in mouse models of liver cancer. Nonviral delivery mechanisms have also demonstrated potential for therapeutic delivery of miRNA. These include liposomes, which have been shown to effectively deliver miRNA to cancer cells locally and systemically in mouse xenograft models. In addition, nanoparticles made of gold or synthetic polymers can effectively deliver miRNA when administered systemically in mouse xenograft models, leading to reduced tumor growth. An exosomal-based miRNA delivery has been reported with great potential as an effective therapeutic delivery system. Exosomal delivery offers a unique opportunity for miRNA delivery because this system is naturally used in cellular communication, probably contributing to its stability in circulation. Exosomes can also be modified to target delivery to a particular cell type. While exosome delivery seems promising, more work must be done to make the production and isolation of exosomes efficient. There is also concern that an immune response could be mounted against exosomes unless they are harvested from immune-compatible cells.

The blood-brain barrier (BBB) is another major challenge for miRNA-based cancer therapy. In addition to brain tumors such as glioblastoma and glioma, several tumor types have brain metastasis at their advanced disease stage. As a result, systemic delivery to the central nervous system (CNS) remains a major obstacle for miRNA-based therapy.^10^

### Tumor suppressor miRNA modification strategies for cancer medicine

Strategies to modify miRNA largely rely on the rich knowledge of ASO chemical modification and small interfering RNA (siRNA) modifications. These include backbone modifications (e.g., phosphorothioates), nucleobase modification, ribose sugar modification, terminal modification, bridged nucleic acids, and bioconjugation (e.g., N-acetylgalactosamine [GalNac]) to improve delivery and pharmacokinetics, pharmacodynamics, and biodistribution.^10^ However, not all modifications are suitable for miRNA, as target specificity and incorporation into the Argonaute (Ago) RNA silencing complex have to be preserved.

miRNA modification represents a novel and promising approach in cancer therapeutics, particularly in pancreatic ductal adenocarcinoma (PDAC).^12^ This approach involves enhancing the stability and efficacy of miRNAs for more effective treatments. One key example is miR-15a, which inhibits cell proliferation and regulates cell cycle control in PDAC. To improve its performance, a modified version, 5-FU-miR-15a, was developed by replacing the uracil residues of the guide strand miRNA with 5-FU. The rationale behind this modification is to combine the therapeutic potential of 5-FU with the tumor-suppressive function of miR-15a.^13^ miR-15a has several important targets such as Bcl2, Wee-1, Chk-1, Bmi-1 and Yap-1, which are significantly overexpressed in PDAC. Additionally, each one of these oncogenic proteins is a therapeutic target for drug development focused on by the biopharmaceutical industry. Notably, 5-FU-miR-15a was shown to maintain its specificity for these targets. Furthermore, once the modified miRNA is degraded within the tumor cells, the released 5-FU molecules were found to exert an additional tumor-killing effect, enhancing the overall therapeutic outcome. This innovative concept was successfully demonstrated in CRC and has shown great potential for PDAC treatment. Notably, 5-FU-miR-15a was found to be potent in inhibiting PDAC metastatic tumor growth and sensitizing PDAC to gemcitabine *in vivo*, showcasing its therapeutic promise. 5-FU-miR-15a was also shown to significantly inhibit the expression of Yap1, Bcl2, interleukin-6 (IL-6), and Mmp9 alone and during treatment with transforming growth factor β1 (TGF-β1) in murine and human pancreatic stellate cells, suggesting reduced proliferation and migration of pancreatic stellate and cancer cells.

Moreover, the strategy of incorporating 5-FU into miR-15a offers a platform technology approach for creating more effective miRNA-based therapeutics for other tumor suppressor miRNAs that are potentially effective in multiple types of cancer, such as leukemia, non-small cell lung cancer, breast cancer, pancreatic cancer, gastric cancer, and CRC.^13^ This approach enhances target specificity and maintains efficacy without needing both *in vitro* and *in vivo* delivery vehicles. The key advantage of this modification is its ability to be delivered to cancer cells vehicle free, which may potentially overcome the challenge of vehicle-associated toxicities (toxicity associated with systemic delivery by lipid-based nanoparticles). This technology platform has broad potential for the development of miRNA-based therapies, and its success is evident in retaining target specificity, enhancing potency, and increasing intracellular stability, making 5-FU-modified miRNA mimetics promising candidates for cancer treatment in the low nanomolar range. The recent clinical results show that 5-FU-miR-15a was well tolerated and showed both clinical and biological activity, as evidenced by disease stabilization in relapsed/refractory (R/R) AML patients from a phase 1 clinical trial (EudraCT number: 2021-006332-46) based on a 5-FU-miR-15a dose-finding and expansion study to investigate the safety, tolerability, and preliminary efficacy of 5-FU-miR-15a (American Association for Cancer Research 2025 Annual Meeting, presentation no: CT-059). There is no immunogenicity in either animal models or patients, suggesting that 5-FU modification may be able to reduce the response from Toll-like receptors (TLRs).

Another platform technology to modify tumor suppressor miRNAs, which shows an enhanced therapeutic efficacy, is the gemcitabine modification of miR-15a.^14^ By replacing the cytidine of the guide strand miRNA with gemcitabine, Gem-miR15a shows significant effects in sensitizing pancreatic cancer to chemotherapy and increased efficacy than its counterpart, unmodified miRNA. In summary, miRNA modification with 5-FU or other chemotherapeutic drugs such as gemcitabine represents an innovative avenue in miRNA-based cancer therapy, paving the way for more effective and targeted treatments with broad applicability in diverse cancer types.

Further investigations into miRNA modifications have shown that various factors can influence the stability of miRNAs, including uridine addition to the miRNA 3′ end and differential processing of pre-miRNAs to mature miRNAs based on secondary structures. These modifications can impact miRNA stability, target specificity, and therapeutic efficacy. Recent studies have utilized a direct link of miRNA (miR-34a) to the folate ligand, FolamiR, to enhance tumor specificity and vehicle-independent delivery.^15^ To enhance stability, the passenger strand of FolamiR-34a was modified with 2′-O-methyl RNA bases to stabilize the miRNA by enhancing nuclease resistance without impairing Ago loading. This approach can effectively deliver miR-34a to tumor cells *in vitro* and *in vivo* with selectivity, as several solid tumors have elevated folate receptors. miRNA can also be conjugated to antibodies specific to tumor antigens to improve tumor cell specificity.

### Future directions

miRNAs offer unique advantages as cancer therapeutics, compared to single-target agents, by suppressing multiple oncogenic targets and pathways. This will be a potential superior feature to overcoming drug resistance, offering major survival benefits and reducing the chance of disease relapse. However, such potential has to be firmly established and justified based on identifying key targets and pathways of the candidate miRNA in a particular cancer type. The oncogenic mRNA targets of miRNA must be elevated significantly in a particular tumor type to have a wider therapeutic window to minimize potential side effects. With the innovative delivery technology and miRNA modification strategies, we are in a critical and exciting time in miRNA-based cancer research and anticancer therapeutic development. In the next 5–10 years, the efforts from academia and industry will pay off to realize the dream of having multi-targeted miRNA-based anticancer therapy to benefit cancer patients. The miRNA-based diagnosis will also impact patient care by offering personalized and tumor-tailored therapy. We remain optimistic about the miRNA-based future medicine in clinical oncology.

## Acknowledgments

This research was supported, in part, by the Veteran Affairs Merit Award, grant number BX005260-01 (J.J.).

## Declaration of interests

J.J. is a founder of Curamir Therapeutics, Inc. and a member of its scientific advisory board. The author has a patent related to this work.
